# Supplementary material for: Neuroprotective effects of essential oils in animal models of Alzheimer’s and Parkinson’s disease: a systematic review
Source: PeerJ. 2025 Jul 10;13:e19643. doi: 10.7717/peerj.19643 (PMC12256039; doi:10.7717/peerj.19643)
Supplement: Supplemental Information 2 [file peerj-13-19643-s002.docx]

**Title:** Essential Oils as a Potential Neuroprotective Role for Neurodegenerative Diseases: Systematic Review"

1. PICOS Search Strategy

PICo: “What is the neuroprotective role of essential oils in models of neurodegenerative diseases?”

|  | **Key-words** | **MESH** | **ENTRY TERMS** |
| --- | --- | --- | --- |
| **P** | Animal models | Animals | Animal models  Model, Animal |
| **I** | Essential oils  Neuroprotection | Oils, Volatile, Neuroprotective Agents | Volatile Oils, Oils, Volatile, Oil, Volatile, Essential Oil, Essential Oils, Neuroprotection, Neuroprotective Effect, Effect, Neuroprotective |
| **Co** | Parkinson’s Disease, Alzheimer’s Disease, Huntington Disease, Amyotrophic Lateral Sclerosis (ALS) | Parkinson Disease, Alzheimer Disease, Huntington Disease, Amyotrophic Lateral Sclerosis | Parkinson's Disease, Parkinson Disease, Disease, Parkinson, Parkinson's, Parkinsons, Alzheimer Disease, Alzheimer's Disease, Disease, Alzheimer's, Alzheimer's, Huntington Disease, Huntington's Disease, Disease, Huntington's, ALS, Amyotrophic Lateral Sclerosis |

| **Database** | **Total articles** |
| --- | --- |
| PubMed | 34 |
| Science Direct | 27 |
| Virtual Health Library | 16 |
| Total | 77 |
| **Duplicates removed** | 30 |
| **Included articles** | 13 |

**Search strategy in PubMed**

**Search 1:** (("Essential Oils"[MeSH] OR "Essential Oils" OR "Oils, Volatile"[MeSH] OR "Volatile Oils") AND ("Neuroprotective Agents"[MeSH] OR "Neuroprotection") AND ("Parkinson Disease"[MeSH] OR "Parkinson's Disease"))

**Search 2:** (("Essential Oils"[MeSH] OR "Essential Oils" OR "Oils, Volatile"[MeSH] OR "Volatile Oils") AND ("Neuroprotective Agents"[MeSH] OR "Neuroprotection") AND ("Alzheimer Disease"[MeSH] OR "Alzheimer's Disease"))

**Search 3:** (("Essential Oils"[MeSH] OR "Essential Oils" OR "Oils, Volatile"[MeSH] OR "Volatile Oils") AND ("Neuroprotective Agents"[MeSH] OR "Neuroprotection") AND ("Huntington Disease"[MeSH] OR "Huntington's Disease"))

**Search 4:** (("Essential Oils"[MeSH] OR "Essential Oils" OR "Oils, Volatile"[MeSH] OR "Volatile Oils") AND ("Neuroprotective Agents"[MeSH] OR "Neuroprotection") AND ("Amyotrophic Lateral Sclerosis"[MeSH] OR "ALS" OR "Lou Gehrig's Disease"))

**Search strategy in Science Direct**

"Essential Oils" AND "Neuroprotection" AND ("Parkinson’s Disease" OR "Alzheimer's Disease" OR "Huntington Disease" OR "Amyotrophic Lateral Sclerosis") Filter by: Subject Areas: Neuroscience, Chemistry. Filter by: Research articles.

**Search Strategy in Virtual Health Library:**

(("essential oils") AND ("neuroprotection")) AND ("Parkinson’s Disease" OR "Alzheimer's Disease" OR "Huntington Disease" OR "Amyotrophic Lateral Sclerosis")
